# Enhancing human milk iodine concentration: a data-driven action plan for Latvia

**DOI:** 10.3389/fnut.2025.1650108

**Published:** 2025-09-24

**Authors:** Līva Aumeistere, Ksenija Kovada, Annamarija Driksna, Jekaterina Kozačenko, Inga Ciproviča

**Affiliations:** ^1^Faculty of Agriculture and Food Technology, Latvia University of Life Sciences and Technologies, Jelgava, Latvia; ^2^Faculty of Medicine and Life Sciences, University of Latvia, Riga, Latvia; ^3^Institute of Public Health, Rīga Stradiņš University, Riga, Latvia

**Keywords:** diet, human milk, iodine, fortification strategies, Latvia

## Abstract

**Background:**

Iodine deficiency remains a significant public health challenge in Europe. However, there is currently a lack of data on iodine status among lactating women and infants in Latvia.

**Methods:**

This cross-sectional study assessed the impact of maternal diet on iodine concentration in human milk. The data obtained were used as reference points for planning targeted interventions to improve iodine supply. Between October 2024 and April 2025, pooled 24-h human milk samples, 72-h food diaries and questionnaires on anthropometric, sociodemographic characteristics were collected from 55 exclusively breastfeeding women residing in Latvia. Human milk iodine concentration was determined using inductively coupled plasma mass spectrometry, while dietary data were analyzed using the Estonian open-access dietary analysis program NutriData.

**Results:**

Human milk iodine concentration among participants varied considerably (15.00–291.00 μg L^−1^) with a median value (86.00 μg L^−1^) below the level indicating optimal iodine supply for an exclusively breastfed infant (i.e., 150 μg L^−1^). A significant positive association was observed between maternal iodine intake and human milk iodine concentration (*ρ* = 0.407, *p* = 0.002). The participants’ diets lacked iodine-rich sources (i.e., fish, dairy, iodized salt) and, without supplementation, did not reach the adequate intake for iodine (i.e., 200 μg per day).

**Conclusion:**

Preliminary data indicate that current maternal dietary practices in Latvia are insufficient to guarantee optimal iodine supply to exclusively breastfed infants. Study results highlight the need for policy reforms and improved nutritional guidance to address the iodine deficiency problem in Latvia.

## 1 Introduction

Iodine is a critical nutrient during infancy, needed for optimal growth and cognitive development of a child (1). Both iodine deficiency and excess can pose risks. Iodine deficiency in early childhood may result in impaired psychomotor development, stunted growth, reduced intellectual development that remains irreversible even with adequate iodine consumption later in life (2). Conversely, excessive iodine intake – though less common – can disrupt thyroid function, particularly in infants with immature thyroid regulation (2). Exclusively breastfed infants solely rely on iodine provided by human milk, but the iodine concentration in human milk depends on the maternal iodine intake. Maintaining optimal iodine levels for lactating women is therefore crucial to safeguard also the infant’s health (2, 3).

Iodine deficiency has been a long-standing public health issue in the European region, including Baltic countries (1, 4). Dietary guidelines recommend that women during the lactation period aim for 200 μg of iodine per day, with a tolerable upper intake level set at 600 μg per day (1). The main sources of iodine in the Nordic and Baltic countries include milk and dairy products, saltwater fish, eggs, iodized table salt and products containing iodized salt, such as bread (1).

Previous research from Latvia has already indicated that lactating women may have insufficient iodine intake, primarily due to low consumption of iodine-rich foods such as fish and dairy products (5). However, these findings were based solely on dietary data, without the use of quantitative biomarkers to assess iodine status. As ~90% of the absorbed dietary iodine is excreted in the urine within 24 h after consumption, urinary iodine concentration has been a widely accepted biomarker for the evaluation of iodine status in different target groups (4). However, during the lactation period, urinary iodine excretion may be proportionally lower as a significant amount (~40–45%) of the dietary iodine is excreted into milk (4, 6–8). Therefore, urinary iodine concentration alone may not correctly reflect iodine status in lactating women, and human milk should also be used as a matrix to measure the concentration of iodine (6, 7). Determination of human milk iodine concentration also allows indirect assessment of iodine supply to exclusively breastfed infants (6).

In populations with mild to moderate iodine deficiency, the adequate daily intake of iodine for infants ≤ 6 months has been set at 90 μg per day (1). The thresholds for human milk iodine concentration are currently uncertain, but Dror and Allen (6) suggest that human milk iodine concentration in the range of 150 μg L^−1^ during the first 6 months would achieve or exceed infant iodine equilibrium and prevent the developmental consequences of iodine deficiency.

This study aimed to obtain preliminary data on human milk iodine concentration among lactating women in Latvia and evaluate current maternal dietary practices regarding iodine intake. The findings are intended to support future research efforts and public health initiatives aimed to ensure optimal iodine provision for lactating women and breastfed infants in Latvia.

## 2 Materials and methods

### 2.1 Study population

This cross-sectional study was conducted during the timeframe – October 2024–April 2025. Women were invited to participate in the research project via targeted ads published on social media (Facebook, Instagram) using Meta Ads that were linked to the research project’s website (9). The inclusion criteria for participation were the following:

- At least 18 years old;
- Have been residing in Latvia for the last 4 weeks (28 days) – meaning have not traveled or lived abroad during this period for work, leisure, or other reasons;
- Last pregnancy was a singleton, full-term (delivery between 37^th^ to 41^st^ week of gestation);
- Infant’s birth weight ≥2,500 g;
- At least 4 weeks (28 days) postpartum and not pregnant again;
- Currently exclusively breastfeeding only one child – an infant that is not older than 6 months;
- No acute illnesses for the mother and infant.

The exclusion criteria were:

- Use of iodine-containing medications;
- Use of synthetic thyroid hormones or antithyroid medications;
- Use of iodine-containing antiseptic solutions and wound dressings in the last 7 days;
- A diagnostic examination with iodinated contrast media in the last 7 days.

### 2.2 Collection of human milk samples, dietary, etc. data

After the woman had completed the electronic informed consent form, the researcher contacted her via e-mail to agree on how to hand over the research materials – in person or via a self-service parcel machine (Omniva Latvia). Research materials included:

- Four graduated polypropylene containers with a screw cap (volume 60 mL, Vacutest Kima, Italy) and one polypropylene container (volume – 50 mL, with a graduated mark for 40 mL, Plastiques Gosselin, France) marked with a unique four-digit code (participant number) for collection of the pooled 24-h human milk sample;
- Human milk sampling instruction, 72-h food diary and questionnaire about anthropometric, sociodemographic, etc. questions in printed or electronic form.

Participants had to choose three consecutive days to fulfill the 72-h food diary and the questionnaire and collect a pooled 24-h human milk sample (on the third day of the study). Participants were instructed to express a few milliliters of milk after feeding the infant. Milk had to be collected from various feeding sessions (morning, mid-day, evening, night). Participants could choose the most convenient method for milk expression (by hand, using a breast pump or combining both methods). Containers with screw caps that were provided could be used to store expressed human milk. During the collection period, containers with collected milk were stored in the refrigerator (~4 °C). In the end, to obtain a pooled sample cooled human milk collected from all expression sessions was poured together. Participants were instructed to mix the milk in each container beforehand to reduce the separation of milk fat that occurs during the refrigeration process. Pooled human milk was poured into the container marked with a four-digit code (participant number). The required minimal volume of human milk for iodine analysis was 10 mL. The container was marked at 40 mL so that participants would not exceed the maximal amount. At the end, the container with pooled human milk sample was put in the household freezer (approximately −18 °C) and the participant contacted the researcher to agree on the most convenient time when the frozen sample can be taken. During sample transportation, a cooler bag with cooling elements was used.

Participants were able to consume a self-chosen diet (no dietary restrictions were applied). To make it easier to fill out the 72-h food diary, study participants had access to an online atlas with portion sizes of different food products, beverages and meals (10). The portion atlas was developed as part of this research project, using portion images from the Estonian open-access dietary analysis program Nutridata (11). The use of images was coordinated with the Department of Nutrition and Sports of the Estonian National Institute of Health Development. Volume measures (handfuls, teaspoons, etc.) could be also used by the participants to indicate portion sizes.

### 2.3 Iodine analysis of human milk samples

Human milk samples for this study were analyzed as routine samples in the laboratory providing testing (Institute of Food Safety, Animal Health and Environment BIOR, Latvia). The concentration of iodine was determined using inductively coupled plasma mass spectrometry system Agilent 7700x (Agilent Technologies, Japan) according to an accredited in-house method BIOR-T-012-195-2018/1. Frozen human milk samples were thawed in hot water (~55 °C) and homogenized using an orbital shaker. Using an automatic pipette 5 mL of the sample was taken to be analyzed in two replicates. Further, 0.5 mL of tetramethylammonium hydroxide was added. To extract iodine compounds, the tubes were sealed tightly, placed in a drying oven and heated for 3 h at 85 °C. After, samples were removed from the drying oven, cooled to room temperature and 1.25 mL of tellurium standard solution (10 mg L^−1^) was added. Then, samples were transferred to 25 mL volumetric flasks and diluted with deionized water (Milli-Q Water Purification System, resistivity value: 18.2 MΩ·cm, Sigma Aldrich, Poland), the concentration of tetramethylammonium hydroxide in the samples reaching 0.5% but the concentration of the tellurium standard solution – 500 μg L^−1^. Then, using a 20 mL syringe, the samples were filtered through a membrane filter with a pore size of 0.45 μm.

The samples were analyzed on the day of preparation. Measurements were performed according to the Agilent 7700x manufacturer’s instructions. The integration time for the isotope 127I was set to 1. The rinse time (Probe Rinse (Sample) and Probe Rinse (Std)) was set to 40. The obtained filtrate was introduced into the nebulizer using a peristaltic pump. Then, the analyzed solution was nebulized and ionized in inductively coupled argon plasma. Ions were introduced through a cone system with high frequency plasma and distributed according to the mass-charge ratio. After distribution, the ions were recorded in the detector, and the obtained signals were evaluated by mass spectrometer software.

Each time the quantitative determination of iodine in the samples was performed, the equipment was calibrated with standard solutions. Iodine concentration was calculated by the following equation:

w=p×c

where:

*w* – iodine concentration in the human milk sample, μg L^−1^;

*p* – determined iodine concentration, μg L^−1^;

*c* – dilution.

### 2.4 Evaluation of dietary data

The 72-h food diaries were analyzed using the open-access dietary analysis program NutriData (11). Information about dietary supplements and meal replacements was taken from the manufacturer’s websites.

### 2.5 Statistical analysis, data visualization

Statistical calculations (median, interquartile range, minimal and maximal values) were performed, and data visualizations were created using Microsoft Office Excel 360. Non-parametric tests, like Spearman’s rank correlation and the Independent-Samples Median test, were applied to evaluate associations between human milk iodine concentration and selected maternal and infant characteristics, as well as dietary factors. These analyses were conducted using International Business Machines Statistical Package for the Social Sciences, version 23. A *p*-value of ≤0.050 was considered statistically significant.

## 3 Results

### 3.1 Characteristics of the participants

Participants represented all statistical regions of Latvia (Riga, Vidzeme, Latgale, Zemgale, Kurzeme), ensuring geographic diversity in the sample. Nevertheless, a substantial proportion of participants were from the capital city, Riga (*n* = 21) or nearby municipalities within the Riga statistical region (*n* = 15). Characteristics of the participants are compiled in the Supplementary Table 1.

### 3.2 Sources of iodine in the participants’ diet

Calculated daily product consumption (data from 72-h food diaries) is compiled in the Supplementary Table 2. Consumption of iodine-rich foods was low. Considering the estimated median daily fish and seafood consumption (9.97 g per day) among the participants, it can be speculated that the fish intake among participants would not reach 300 to 450 g per week (ready-to-eat weight), that is recommended by the Nordic Nutrition Recommendations (1). Also, milk and dairy product intake among the participants was observed to be lower (on median – 242.72 g per day) than recommended by dietary guidelines (i.e., 350–500 g per day) (1). A higher dietary iodine intake was associated with the consumption of milk and dairy products (*ρ* = 0.679, *p* < 0.0005), eggs (*ρ* = 0.593, *p* < 0.0005), fish and seafood (*ρ* = 0.324, *p* = 0.016), but lower – with the consumption of potatoes (*ρ* = −0.335, *p* = 0.013) and plant-based product alternatives (*ρ* = −0.269, *p* = 0.047).

Some of the participants reported that they have eliminated or reduced the intake of certain food products in their diet, most commonly – milk and dairy products (*n* = 12, 22%). Dietary restrictions were most often aimed at improving the infant’s health (cow’s milk protein allergy, colic, etc.). Only eight participants (15%) noted using iodized salt in the household.

**Figure 1:**
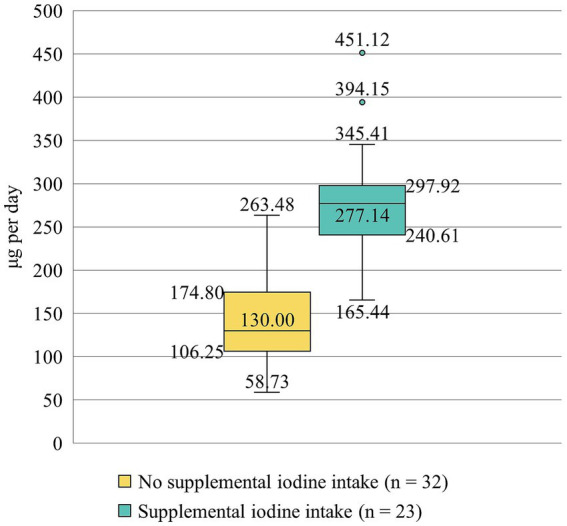
Daily iodine intake among participants (μg per day) without and with supplemental iodine intake. The box plots display the median, interquartile range, and full range of calculated iodine intake values. Outliers are indicated as individual points.

The median iodine intake from dietary sources was 126 μg per day. Only 15% of participants (*n* = 8) were able to achieve adequate iodine intake from diet alone (i.e., at least 200 μg of iodine per day) (1). Twenty-three participants reported consuming dietary supplements, meal replacements containing iodine (Table 1). Iodine intake was significantly higher among those with supplemental intake (*p* < 0.005) (Figure 1). There was no difference in sociodemographic characteristics (educational degree, income, etc.) between participants using supplementation versus those not using iodine supplementation (*p* > 0.05). None of the participants exceeded the tolerable upper intake for iodine (i.e., 600 μg per day) (1).

**Table 1:** Supplemental iodine sources among the participants (*n* = 23).

Supplemental iodine source	Number of participants	Daily supplemental iodine intake
Monopreparate containing potassium iodine	1	100 μg per day
Monopreparate containing brown seaweed	1	150 μg per day
Complex dietary supplement containing potassium iodine	20	Varied between participants from 75 to 200 μg per day
Complex dietary supplement + various meal replacements, all containing potassium iodine	1	267 μg per day for the first and third day of the study 442 μg per day for the second day of the study

### 3.3 Iodine concentration in human milk samples

**Figure 2:**
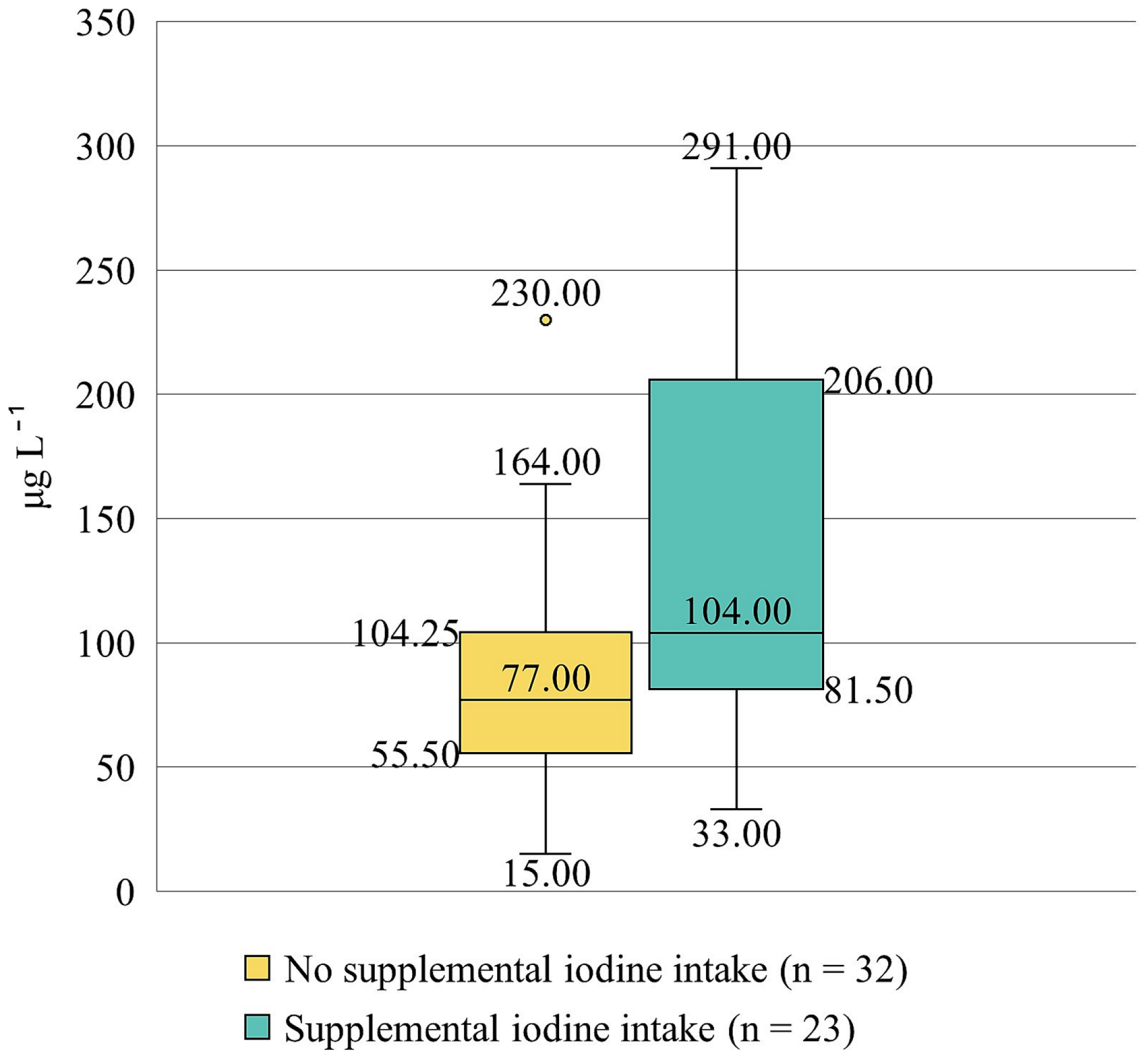
Human milk iodine concentration (μg L⁻^1^) among participants without and with supplemental iodine intake. The box plots display the median, interquartile range, and full range of human milk iodine concentration levels. An outlier is indicated as an individual point.

Iodine concentration in human milk varied widely among study participants – from 15.00 to 291.00 μg L^−1^, with median concentration reaching 86.00 μg L^−1^. Only about one in five participants (*n* = 12, 22%) had human milk iodine concentration at the level that would indicate optimal iodine supply to an exclusively breastfed infant (i.e., ≥150 μg L^−1^) (6). A significant positive association was observed between maternal iodine intake and human milk iodine concentration (*ρ* = 0.407, *p* = 0.002) and, overall, a higher human milk iodine concentration was observed among the participants with supplemental iodine intake (*p* = 0.027) (Figure 2). A higher human milk iodine concentration was associated with the consumption of fish and seafood (*ρ* = 0.351, *p* = 0.009), but lower – with the consumption of plant-based product alternatives (*ρ* = −0.337, *p* = 0.012). None of the assessed infant-related characteristics – including birth weight, age, and sex – or maternal characteristics such as age, current body mass index, etc. showed a statistically significant association with iodine concentration in human milk (*p* > 0.05).

## 4 Discussion

This is the first study to report data on human milk iodine concentration in the Baltic region, and the obtained results are similar to the values reported from the Nordic countries, like Norway (68 μg L^−1^), Sweden (90 μg L^−1^), Iceland (84 μg L^−1^) (12–14). With median human milk iodine concentration (86 μg L^−1^) not reaching the level that would indicate optimal iodine supply to an infant (i.e, ≥150 μg L^−1^) (6), our results clearly demonstrate the problem of iodine deficiency in Latvia and create the need for action. Therefore, in the following sub-sections, we contextualize our research findings, discuss the influencing factors, and outline potential strategies for improving iodine supply among lactating women and breastfed infants, as well as in the overall population of Latvia.

### 4.1 Promotion of iodized salt

Although the World Health Organization states that salt iodization is the main strategy to ensure adequate iodine intake in the European region (4), salt iodization in Latvia is still voluntary. Only a few producers offer salt with added iodine, in contrast to the wide range of other edible salt available in stores in Latvia (rock, sea, Himalayan pink, black, spice salt, salt with reduced sodium content, etc.) (15). Also, rarely did participants of this study (*n* = 8) report consuming iodized salt. To encourage people to prefer iodized salt, stores should enhance in-store visibility and placement, highlighting iodized salt on food shelves, as well as providing other iodized salt promotion activities. However, the overall salt consumption in Latvia is excessive (15). Therefore, activities to promote iodized salt must be planned and implemented in a way that does not increase the total salt consumption in society.

### 4.2 Promotion of iodine-rich foods

Data from this study indicate that including fish and seafood in the diet ensures a higher human milk iodine concentration. To promote the consumption of fish and seafood, firstly, it is necessary to evaluate and analyze the reasons why those products are only rarely consumed. Depending on the main reasons (price, sensory properties, lack of availability in stores, etc.), it will be possible to implement the most appropriate interventions to increase fish and seafood intake among lactating women in Latvia.

High iodine content in animal feed could be the reason why Latvia stands out among other European countries with the highest reported iodine concentration in milk from dairy cows (499 μg kg^−1^) (4), and the data from this study confirm that milk and dairy products plays a significant role in the iodine intake for lactating women. Therefore, interventions should be emphasized on the popularization of milk and dairy products as a valuable source of iodine to increase the uptake.

### 4.3 Food reformulation

In 2022, a memorandum of cooperation on improving the composition of food products was signed between the Latvian Ministry of Health, Federation of Food Enterprises and Chamber of Commerce and Industry (16). Taking into account experience from Belgium, where an agreement was signed between the bakery sector and the Ministry of Health, to fortify bread with iodized salt (17), the first step to optimize iodine intake in Latvia could be to supplement the aforementioned memorandum by additional criteria to offer producers a voluntary commitment to reformulate certain food products by increasing iodine content in them. One of these products could be bread, as this product was part of the daily diet for most participants in this study.

Lower iodine concentration in human milk was linked to greater consumption of plant-based alternatives, as some participants had reduced or eliminated milk and dairy products, replacing them with plant-based options, mostly plant-based drinks added to the coffee as an alternative to the milk. Sea salt is commonly added to plant-based drinks available in Latvia. Although the name indicates that sea salt could be a good source of iodine, unless it is iodized, the iodine content in sea salt and, therefore, in plant-based drinks is negligible (11, 15). The World Health Organization (4) also has pointed out that the increasing popularity and availability of plant-based alternatives to key sources of iodine, such as milk, dairy products and fish, is contributing to persistent and increased insufficient iodine intake in the European Region and urgently calls for iodine fortification of plant-based alternatives.

### 4.4 Improving the dietary knowledge of healthcare specialists

Preliminary results of this study have already been applied to promote dietary knowledge of healthcare specialists. In April 2025, proposals were submitted for the Maternal and Child Health Improvement Plan 2025–2027 (18), to include a task to update guidelines for healthcare professionals advising women on healthy nutrition during pregnancy and the lactation period (19). The revised guidelines will provide standardized recommendations on iodine and other dietary supplementation, as well as include recommendations for maintaining optimal iodine status if a woman follows an exclusion diet (dairy-free diet, vegan diet, etc.).

## 5 Limitations

The number of human milk samples analyzed was small. However, the data indicate a trend that iodine status among lactating women and exclusively breastfed infants in Latvia may not be optimal. For a more accurate evaluation, in addition to human milk samples, urine samples from both mothers and infants should also be collected for the determination of iodine concentration.

Although human milk samples were collected at home, participants were instructed on how to correctly collect a pooled human milk sample. Given the study population – new mothers – this method was the most feasible and participant-friendly, without compromising the reliability of iodine measurements.

As there is currently no freely available national food composition database in Latvia, the NutriData program (11) from the neighboring country – Estonia was used. Its database covers over 4,600 commonly consumed foods, including many Latvian products, with each nutrient entry sourced and method-tagged for reliability.

While the 72-h food diary is well-suited to capturing recent iodine intake, which most directly reflects short-term fluctuations in human milk iodine concentration, it may not fully represent habitual dietary intake of iodine. For future research, the use of an iodine-specific food frequency questionnaire could provide complementary insights by estimating longer-term iodine consumption patterns and help to identify women at risk of inadequate iodine intake.

The proposed interventions for improving iodine supply among lactating women and breastfed infants, as well as in the overall population of Latvia, currently remain theoretical. Practical implementation and evaluation are needed to determine which strategies are the most effective.

## 6 Conclusion

Preliminary data indicate that current maternal dietary practices in Latvia are insufficient to guarantee optimal iodine supply to exclusively breastfed infants. Study results highlight the need for policy reforms and improved nutritional guidance to address the iodine deficiency problem in Latvia.

## Data Availability

The datasets presented in this study can be found in online repositories. The names of the repository/repositories and accession number(s) can be found: Latvian National Research Data Repository Dataverse.lv. doi.org/10.71782/DATA/D2OABD.
