# Supplementary material for: Enhancing human milk iodine concentration: a data-driven action plan for Latvia
Source: Front Nutr. 2025 Sep 24;12:1650108. doi: 10.3389/fnut.2025.1650108 (PMC12504860; doi:10.3389/fnut.2025.1650108)
Supplement: Supplementary file 1 [file Table_1.DOCX]

**Supplementary Table 1. Characteristics of the participants (n = 55)**

| **Characteristics** | **Median [interquartile range] (minimal–maximal value)** | | | | | |
| --- | --- | --- | --- | --- | --- | --- |
| Maternal age (years) | 31 [7] (23–40) | | | | | |
| Current body mass index (kg m^-2^)^[[1]](#footnote-1)^ | 23.78 [3.5] (17.92–31.60) | | | | | |
| Weight gain during pregnancy (kg) | 13 [5] (0–30) | | | | | |
| Age of the infant (months) | 4 [2.5] (1–6) | | | | | |
| **Characteristics** | **Category (count, percentage)** | | | | | |
| Infant’s birth weight (g) | 2500–3999 (n = 45, 82%) | | 4000–4499 (n = 7, 13%) | | ≥4500 (n = 3, 5%) | |
| Nationality | Latvian (n = 52, 95%) | | | Other (n = 3, 5%) | | |
| Educational degree | Upper Secondary (n = 2, 4%) | College (n = 4, 7%) | | Unfinished Higher (n = 7, 13%) | | Higher (n = 42, 76%) |
| Total household income after tax (Eur) | No information provided (n = 5, 9%) | ≤2000 (n = 15, 27%) | | 2001–3000 (n = 16, 29%) | | ≥3001 (n = 19, 35%) |
| Delivery mode | Vaginal (n = 38; 69%) | | | Caesarean (n = 17; 31%) | | |
| Sex of the infant | Boy (n = 28, 51%) | | | Girl (n = 27; 49%) | | |
| Smoking | No (n = 55, 100%) | | | Yes (n = 0, 0%) | | |
| Chosen human milk sample collection method | By hand (n = 14, 25%) | | Breast pump (n = 34, 62%) | | Both methods (n = 7, 13%) | |

1. Body mass index calculation was performed based on weight and height values reported by the participant. No anthropometric measurements were performed during this research. [↑](#footnote-ref-1)
