# Supplementary material for: Enhancing human milk iodine concentration: a data-driven action plan for Latvia
Source: Front Nutr. 2025 Sep 24;12:1650108. doi: 10.3389/fnut.2025.1650108 (PMC12504860; doi:10.3389/fnut.2025.1650108)
Supplement: Supplementary file 2 [file Table_2.DOCX]

**Supplementary Table 2. Product consumption among the participants, g per day (n = 55)**

| **Product category** | **Minimal value** | **25^th^ percentile** | **Median** | **75^th^ percentile** | **Maximal value** |
| --- | --- | --- | --- | --- | --- |
| **Cereal products** – **dry ingredients, pseudograins, corn products** | 2.13 | 52.19 | 87.75 | 111.86 | 254.33 |
| **Bread products** | 0.00 | 35.83 | 61.68 | 92.83 | 185.67 |
| **Potatoes** | 0.00 | 0.00 | 50.00 | 133.96 | 250.10 |
| **Vegetables (fresh, frozen, dried, cooked, canned)** | 16.67 | 136.84 | 194.31 | 315.17 | 597.48 |
| **Fruits and berries (fresh, frozen, dried, cooked, canned)** | 0.00 | 121.50 | 200.00 | 313.33 | 757.00 |
| **Milk, dairy products** | 0.00 | 110.71 | 242.72 | 358.32 | 698.17 |
| **Plant-based product alternatives** | 0.00 | 0.00 | 0.00 | 91.03 | 336.67 |
| **Meat, offal and meat products** | 0.00 | 96.98 | 144.35 | 207.10 | 558.86 |
| **Fish and seafood** | 0.00 | 0.00 | 9.97 | 44.72 | 188.77 |
| **Eggs** | 0.00 | 8.08 | 43.07 | 72.21 | 193.03 |
| **Fats** | 7.30 | 20.64 | 48.43 | 66.98 | 289.11 |
| **Sugar, honey, confectionery, dessert sauces** | 0.00 | 36.70 | 62.39 | 98.32 | 272.80 |
| **Pastry and bakery products** | 0.00 | 0.00 | 13.33 | 35.17 | 100.00 |
| **Savory snacks** | 0.00 | 0.00 | 0.00 | 8.33 | 66.67 |
| **Water** | 250.67 | 1246.96 | 1839.28 | 2155.02 | 3961.67 |
| **Other non-alcoholic beverages** | 0.00 | 274.75 | 500.00 | 790.00 | 1953.33 |
| **Alcoholic beverages** | 0.00 | 0.00 | 0.00 | 0.00 | 0.00 |
